# Polyploidization Altered Gene Functions in Cotton (*Gossypium* spp.)

**DOI:** 10.1371/journal.pone.0014351

**Published:** 2010-12-16

**Authors:** Zhanyou Xu, John Z. Yu, Jaemin Cho, Jing Yu, Russell J. Kohel, Richard G. Percy

**Affiliations:** Crop Germplasm Research Unit, Southern Plains Agricultural Research Center, United States Department of Agriculture-Agricultural Research Service (USDA-ARS), College Station, Texas, United States of America; Montreal Botanical Garden, Canada

## Abstract

Cotton (*Gossypium* spp.) is an important crop plant that is widely grown to produce both natural textile fibers and cottonseed oil. Cotton fibers, the economically more important product of the cotton plant, are seed trichomes derived from individual cells of the epidermal layer of the seed coat. It has been known for a long time that large numbers of genes determine the development of cotton fiber, and more recently it has been determined that these genes are distributed across At and Dt subgenomes of tetraploid AD cottons. In the present study, the organization and evolution of the fiber development genes were investigated through the construction of an integrated genetic and physical map of fiber development genes whose functions have been verified and confirmed. A total of 535 cotton fiber development genes, including 103 fiber transcription factors, 259 fiber development genes, and 173 SSR-contained fiber ESTs, were analyzed at the subgenome level. A total of 499 fiber related contigs were selected and assembled. Together these contigs covered about 151 Mb in physical length, or about 6.7% of the tetraploid cotton genome. Among the 499 contigs, 397 were anchored onto individual chromosomes. Results from our studies on the distribution patterns of the fiber development genes and transcription factors between the At and Dt subgenomes showed that more transcription factors were from Dt subgenome than At, whereas more fiber development genes were from At subgenome than Dt. Combining our mapping results with previous reports that more fiber QTLs were mapped in Dt subgenome than At subgenome, the results suggested a new functional hypothesis for tetraploid cotton. After the merging of the two diploid *Gossypium* genomes, the At subgenome has provided most of the genes for fiber development, because it continues to function similar to its fiber producing diploid A genome ancestor. On the other hand, the Dt subgenome, with its non-fiber producing D genome ancestor, provides more transcription factors that regulate the expression of the fiber genes in the At subgenome. This hypothesis would explain previously published mapping results. At the same time, this integrated map of fiber development genes would provide a framework to clone individual full-length fiber genes, to elucidate the physiological mechanisms of the fiber differentiation, elongation, and maturation, and to systematically study the functional network of these genes that interact during the process of fiber development in the tetraploid cottons.

## Introduction

*Gossypium*, composed of 50 species including 45 diploid and 5 allopolyploid species[1], is an excellent system for studying many fundamental questions relating to genome evolution, plant development, polyploidization, and crop productivity. The diploid *Gossypium* species have been grouped into eight cytological genomes, designated A through G, and K[2], [3], with the same chromosome number (n = 13). Among diploid cottons, only the A-genome species produce spinnable fibers (seed trichomes), although there are genes reported to relate to fiber development in the Dt subgenome of tetraploid cotton species n = 26 [4], [5], [6], [7], [8], [9]. Only four species of *Gossypium* are cultivated: two “New World” tetraploid species, *G. hirsutum* and *G. barbadense*, and two “Old World” diploid species, *G. arboreum* and *G. herbaceum*. The A and D genomes are estimated to have diverged from a common ancestor between 6 and 11 million years ago (*MYA*) [10], and the “New World” tetraploid species arose some 1–2 *MYA* through the hybridization of the A genome and the D genome [11], [12].

Trichomes are unicellular or multicellular appendages originating from cells of the aerial plants [13], and are functionally classified as glandular vs. non-glandular trichomes [14], [15]. Morphologically, trichomes are either branched or non-branched [16]. Cotton fibers, produced only by certain species in the genus *Gossypium*, are non-glandular, non-branched seed trichomes consisting of extremely elongated single cells derived from the epidermal layer of the seed coat [17]. Thus cotton fiber is a model system to study single cell differentiation, development, and maturation of other plants cells. Cotton fiber development is a complex process and the fiber transcriptome represents 35–40% of the genes in the cotton genome [18], assuming that the total number of genes in the cotton genome is approximately 53,000 [19]. Spinnable cotton fiber development is delineated into four discrete but overlapping developmental stages: fiber initiation, elongation, secondary wall biosynthesis, and maturation [17], [20], [21]. The number and weight of the cotton lint fibers vary with the levels of hormones present [22] and with temperatures occurring during development [23]. However, under normal developmental conditions, only about one third of all the epidermal cells become fibers [24]. A better understanding of the genetic processes that regulate which and how many epidermal cells become fibers and the genetic processes that regulate fiber elongation would allow us to biologically manipulate the single cells to increase yield and improve fiber length and uniformity for a higher quality fiber. Both cotton scientists and other plant biologists have focused on the isolation, characterization, and evaluation of genes related to fiber development [25].

Knowledge and understanding of genes, regulatory factors, specific promoters, and biochemical processes of fiber growth and development has increased greatly over past decades. Many individual fiber genes were cloned and characterized prior to the widespread use of microarray technology[26], [27], [28], [29], [30], [31], [32], [33], [34]. Numerous investigations on transcription factors that regulate the development of cotton fibers have occurred [29] [35] [36] [37] [38], but none successfully localize the expression of genes specifically to cotton fiber development. There also have been large numbers of genes and regulatory factors related to cotton fiber development that have been isolated, functionally investigated by microarray and RT-PCR, and comparatively annotated with Arabidopsis trichome genes [8], [39], [40], [41], [42], [43], [44]. Different studies on individual fiber genes isolated from various sources and characterized by different methods all emphasized that their identified gene(s) play a very important role during fiber development. However, these genes may be at only a point on the long physiological pathway. Further, no efforts have been made to determine the bottleneck step or steps and their correspondent gene(s) or protein(s) in the physiological pathway of fiber development.

With the exception of a single transcription factor, GhMYB109, no report or systematic study has been made to anchor fiber genes in the cotton genome, to study their genome-wide distribution, organization, evolution, and interactions. The exception, GhMYB109, was characterized as a single-copy gene in the cotton genome by Southern blot analysis [29]. There has been some reports, based upon numbers of identified fiber genes or QTLs, that contributions of the Dt subgenome are more significant to fiber development than those of the At subgenome [6], [9], although the D genome ancestor does not produce fiber. On the other hand, some reports suggested that the At subgenome was more important than Dt subgenome [4], [38], [45]; while other reports argued that they were equally important for fiber development [41], [42], [46]. Even from the same research group, conflicting results were reported that more fiber EST-derived eSSRs were mapped in Dt subgenome than At [40], or in contrast, more EST-SSRs were mapped in At than Dt from same mapping population [41].

The reasons for the inconsistency may be due to both limited numbers of available markers and non-random markers used in the analysis. In order to resolve these conflicts and to elucidate the distribution, organization, and network of genes for fiber development in tetraploid cottons, a total of 535 fiber-related genes of known function in fiber development, including both fiber development genes and transcription factors, were collected and anchored to integrated genetic and physical contig maps. The distribution and organization of these genes were analyzed and the results showed that more transcription factors were from the Dt subgenome than the At subgenome, whereas more fiber development genes were from the At than the Dt. Based on these results and previous reports, the data suggested a functional hypothesis for allotetraploid cotton that has resulted from the merger of two diploid *Gossypium* genomes, in which the At subgenome is functionally similar to its fiber producing A genome diploid ancestor. In the resulting allotetraploid the At subgenome provides most of the genes for fiber development. On the other hand, the Dt subgenome, with its D genome ancestor that did not produce fiber, provides more transcription factors that regulate the expression of the fiber genes in the At subgenome. The transcription factors anchored only in Dt subgenome function as neofunctionalization [47], factors shared by both At subgenome and Dt function as subfunctionalization [47]. This hypothesis would explain previously published results.

## Results

### Assembly of fiber genes into sequence contigs

A total of 535 fiber development genes and transcription factors (Supplemental Table S1) were collected from previous published reports and their sequences were downloaded from NCBI (http://www.ncbi.nlm.nih.gov/). Among them, 259 were fiber development genes, 103 were transcription factors, and 173 were genetically mapped SSR-contained fiber ESTs. Individual genes were assembled into sequence contigs for three main reasons: the first was to remove redundancy of all the sequences for the subsequent Overgo primer design; the second was to crosscheck the functions of the assembled genes in each contig; and the third was to further link assembled contigs/singletons with other sequence-tagged-sites (STS), which included BAC-end sequences, BAC sub-clone sequences, and mapped genetic marker sequences in the integrated genetic and physical map of tetraploid cotton. A total of 448 unique sequences were obtained from an assembly of 535 sequences by Sequencher V4.2 (http://www.genecodes.com/), including 46 sequence contigs (Table 1) and 402 sequence singletons. Annotation of the individual genes was cross-verified from their sequence contigs, and functions of the 46 sequence contigs were summarized according to the original annotation of the individual genes. Most annotations (40 of the 46) of the sequence contigs were consistent with their original function analysis. Such contig as “Scontig02” has three gene fragments or transcription factors, a RD22-like protein, GhRDL, from *G. hirsutum* [37], a GaRDL1 from *G. arboreum* [35], an up-regulated elongation gene, P3D11, from *G. hirsutum* [48], and a promoter (RDL-P3) from *G. arboreum* [35]. Their functions were similar and they were assembled as a contig. Only two of 46 contigs contained differently annotated genes. For example, Scontig19 has two gene fragments, one was annotated as auxin-binding protein GhABP [48], and the other was annotated as cotton-fiber germin-like protein GhGLP1 [28]. Three contigs have identical functional annotations, and they are marked as “r” in the Table 1. Scontig28 was a typical contig that had one long annotated full length arabinogalactan protein mRNA sequence (GenBank accession # ay218846) and five overlapped short sequences (Table 1). The 428 unique sequences assembled by “Sequencer” were further verified by DNA assembly software “CAP3” (http://pbil.univ-lyon1.fr/cap3.php) [49], and no further contigs were obtained.

**Table 1:** List of the 46 gene sequence contigs, their sizes and functions in fiber development.

Contig name*	Size (Bp)	Seq. no.	FCV (y/n/r)	Functionannotation
Scontig01	2,106	14	Y	Actin gene & up-regulated elongation gene
Scontig02	1,570	4	Y	RDL promoter and up-regulated elongation gene
Scontig03	1,087	4	R	Two polypeptide for elongation not initiation
Scontig04	354	2	Y	MYB transcription factor for initiation
Scontig05	484	3	Y	Up-regulated elongation gene
Scontig06	462	2	Y	Up-regulated elongation gene
Scontig07	232	2	Y	Up-regulated elongation gene
Scontig08	799	2	Y	Up-regulated elongation gene
Scontig09	2,694	3	Y	HOX3 homeodomain protein (transcription factor)
Scontig10	1,865	4	Y	3-ketoacyl-CoAsynthase & up-regulated gene for elongation
Scontig11	1,787	2	Y	Serine carboxypeptidase for initiation & elongation
Scontig12	2,436	4	Y	Actin gene for initiation & elongation
Scontig13	1,401	2	Y	Glucuronosyl tranferase gene for elongation
Scontig14	1,567	2	Y	translation elongation factor 1A1 & A2
Scontig15	357	4	Y	Up-regulated elongation gene
Scontig17	558	2	Y	Up-regulated elongation gene
Scontig19	953	2	N	Auxin-binding protein(ABP) for elongation Cotton-fiber germin-like protein for elongation
Scontig20	452	2	Y	Up-regulated elongation gene
Scontig23	1,294	2	Y	Alpha-expansin precursor for elongation
Scontig24	5,765	2	Y	b-tubulin protein for elongation
Scontig25	395	2	Y	Up-regulated elongation gen
Scontig26	407	2	Y	Up-regulated elongation gene
Scontig27	484	3	Y	Up-regulated elongation gene
Scontig28	1,206	6	R	Arabinogalactan protein for elongation (consistent)
Scontig29	749	2	Y	Up-regulated elongation gene
Scontig31	1,250	4	Y	Expansin gene for elongation (AY189969) expansin gene for elongation only (pGhEX1) Up-regulated elongation gene (PCC08 & PC = 1C12)
Scontig32	1,638	4	Y	Beta-tubulin geneUp-regulated elongation gene
Scontig34	2,632	2	R	Sucrose synthase gene (Ruan et al., 1998) Sucrose synthase gene (Wu, YR et al., 2006)
Scontig35	1,875	7	Y	myb transcription factorselongation candidate gene
Scontig36	1,476	2	Y	Putative acyltransferaseUp-regulated elongation gene
Scontig37	1,738	2	R	Translation elongation factors for elongation
Scontig42	884	2	Y	Up-regulated elongation gene
Scontig44	345	2	Y	Up-regulated elongation gene
Scontig45	893	3	Y	Elongation gene candidate
Scontig46	787	2	Y	Hypothetical protein for elongationUp-regulated elongation gene
Scontig48	668	3	Y	Up-regulated elongation gene
Scontig49	573	2	Y	Up-regulated elongation gene
Scontig50	817	2	Y	Tubulin/elongation gene candidate
Scontig51	924	2	Y	Ga & Gh MYB109 transcription factor
Scontig52	293	2	Y	Elongation gene candidate
Scontig54	924	2	Y	MYB-like DNA-binding domain protein 2 mybfamilytranscriptionfactor2/fiberfactor1
Scontig55	592	2	Y	Elongation gene contains initiation gene
Scontig56	1,283	2		Transcription factor GhMYB25
Scontig57	1,379	2	Y	xyloglucan endotransglycosylase gene
Scontig58	804	2	Y	Up-regulated elongation gene
Scontig62	1,501	2	N	ARF transcription factorsElongation gene candidate
Total	54,740	132		

*Scontig for sequence-based contigs. FCV for Function Consistency verification of the gene fragments in the contigs, y  =  yes, n  =  no, and r  =  repeated gene sequence.

### Assembly of sequence contigs with STS markers mapped to integrated map of tetraploid cotton

STS markers are sequence-tagged sites whose location and base sequence are known in the genome. They are useful for localizing and orientating the sequence data, and serve as landmarks on the physical map of a genome. In the current integrated genetic and physical map of tetraploid cotton, there are 10,416 STS markers, including 3,614 BAC-end sequences, 6,152 genetic loci with whole fragment sequences, and 750 sub-clone sequences (Xu et al., under preparation). Gene sequences (259) and transcription factors (103) with a total of 362 sequences were used to assemble sequence contigs with 10, 416 STSs to anchor the unmapped genes in the integrated map. Only three (Table 2) of the 362 sequences (0.8%) were assembled into contigs (Gene-ctg10, 22, and 45) and anchored in the integrated map at a higher stringency (overlap 50 bases at minimum match >90%) than that for Overgo hybridization (40–44 bases). The reasons that less than 1% of the genes were mapped by sequence contig assembly between the fiber gene sequences and STSs were because: 1) few STSs were mapped compared with a total gene number of 53,000, even if all the 10,416 STSs in the map were genes, its coverage of the genome only accounted for about 19.6%, in fact, most of the STSs in the integrated map of tetraploid cotton were not functional genes, but mapped genomic sequences; 2) sequence contig assembly only detected overlapped sequences that link end by end, and it is different from blast analysis that compares the sequences not only at the two ends of the sequences, but also throughout the whole sequences; and 3) the collected genes were relatively new and not mapped in previous published maps. In general, it is not practical to anchor unmapped genes or sequences by contig assembly with STS sequences because of both the limited number of the STSs in the integrated map and the end-by-end detection rules of sequence contig assembly.

**Table 2:** List of the anchored fiber development genes/transcription factors assembled with STS markers and their functions and locations in the tetraploid cotton genome.

Fiber gene Name	Gene/Factors	STS name	Function annotated	Location in Genome	Overlapped base number
Gene-ctg10	Gene-GhEF1A2	COAU0001M07	Fiber elongation	Chr.01-[97.3]	559
Gene-ctg22	Gene-P2B08	Gate4DB11	Fiber elongation	Chr.26-[114.1]	431
Gene-ctg45	f-DT544876	CBV028F22_R	Heat stress transcription factor	Chr.26-[92.1]	549

### Screening BAC/BIBAC libraries and assembling the positive clones

#### BAC library screening

A total of 448 unique gene sequences were used to design Overgo primers through Overgo designer V1.02 (http://www.mouse-genome.bcm.tmc.edu//webOvergo/OvergoInput.asp), and 440 (98.2%) Overgo probes were obtained. Of the 440 Overgo probes, 396 identified positive BAC clones that accounted for up to 90% of all the Overgo probes, and this result indicated that the two BAC libraries used for BAC screening may cover about 90% of the fiber development related genes of the tetraploid cotton genome. A total of 1,865 positive clones were identified from two BAC libraries representing a 9.7×haploid coverage of the chromosomes [50]. On average, there were 5.6 positive clones for each Overgo primer, which is much lower than the 9.7 x genome coverage estimate of the two BAC libraries. The reasons for the low coverage of positives may be because: first, all the probes are from ESTs or genes and most of them have fewer copies or even a single copy in the genome; and second, some of the low-copy or single-copy genes are in genome locations that are difficult to clone. After the two-round hybridization selection (details see Materials and Methods) and comparison with the genome-wide physical contig map, a total of 5,005 positive clones were identified for fingerprinting.

#### BAC fingerprinting and contig assembly

An initial total of 5,005 positive clones identified from the BAC libraries were fingerprinted and the raw data was edited into FPC format via software “GenoProfiler” [51]. From the total number of clones, 170 clones (3.4%) were removed following fingerprint editing because they either failed in fingerprinting or had small inserts with no digestion. In addition, 81 clones (1.6%) were ignored by the FPC [52] program during contig assembly because they contained five or fewer bands providing insufficient information to be included in the contig assembly. Thus, a final total of 4,754 clones were successfully fingerprinted and integrated into the FPC database.

The FPC database of 4,754 BAC fingerprints was subjected to contig analysis using FPC software. The parameters, cutoff ranges 1e−25 to 1e−10 and a tolerance of band 0.2 bp, were employed for the contig assembly. After manual editing and merging, 499 BAC contigs and 17 singletons were obtained (supplemental Table S2). The average number of DNA bands generated from each clone was 40 on a calculation from the 4,754 FPC database. On average, each band counted for approximately 3,525 bp, based on an overall average insert size for the three libraries of 141 kb [50]. There were 42,970 unique bands in the contigs and the total physical length of contigs was estimated to be 151,469 Mb with an average of 304 kb per contig. Based on an estimated genome size 2,250 Mb of *G. hirsutum*, the coverage of the 499 contigs accounted for 6.7% of the tetraploid cotton genome.

### Anchoring of the contigs to a genetic map

**Figure 1:**
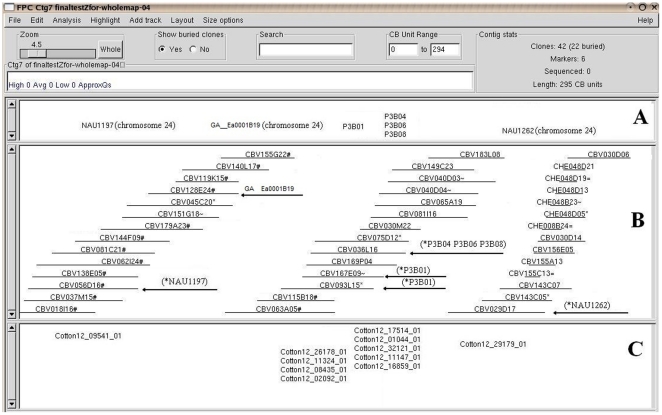
Integrated physical and genetic map of contigs 7. Example of a tetraploid cotton BAC/BIBAC contig anchored to Dt subgenome chromosome 24. This contig consisted of 42 clones from two source libraries and was estimated to span 1,032 kb. This integrated contig contains three parts: Part A is the genetic markers (NAU1197 and NAU1262, [40]), EST (GA__Ea0001B19, [44]), and five development genes (P3B01, P3B04, P3B06, and P3B08, [48]) that were anchored to contigs by Overgo hybridization; Part B is the overlapped continued BAC clones, the contig. The arrow indicated clone is the positive clone of the marker or fiber genes; Part C is the EST Unigenes that were anchored to contigs by both Overgo hybridization and sequence comparison, same unigene names were used as original paper (Udall et al., 2006). The clone names contain 9 characters, the first three letters are library name (CBV stands for **C**otton ***B*** *am*HI, and vector **V**04541; CHE stands for **C**otton ***H*** *in*d III vector p**E**CBAC1), the following three digits are the microtitter plate number, and the last three characters are the clone position in a microtitter plate. Such as “CBV056D16”, it means that this clone came from cotton TM-1 *Bam*HI, vector V04541 library, and located in D row column 16 in microtiter plate number 56.

The 499 identified BAC contigs were anchored to chromosomes by genetic markers. Of the 499 contigs, 381 contigs were anchored to the 26 chromosomes of tetraploid cotton, 102 contigs without a genetically mapped marker could not be integrated into genetic map, and 16 contigs were anchored to diploid D (*G. raimondii*) genome only. Of the 381 mapped contigs, 135 (35.4%) were located in At subgenome only, 89 (23.4%) were located in Dt subgenome, and 157 (41.2%) of them were shared between At and Dt subgenomes (Table 3). Percentage (41.2%) of shared genes between subgenomes At and Dt from this report is consistent with the results (42.3%) obtained by comparing all the 51,107 EST unigenes in two subgenomes (Xu et al., 2008). Percentages of the genes in At and Dt subgenomes is very close by 35.4% and 23.4%, respectively. As an example of the integrated contigs, ctg0007 in figure 1 demonstrates how the contig was anchored to an individual chromosome.

**Table 3:** Distribution patterns of fiber development genes and transcription factors in AD tetraploid cottons.

Subgenome/genome origination	No. of contigs from 103 transcription factors	No. of contigs from 173 SSR- containing fiber ESTs	No. of contigs from 259 fiber development genes	No. of contigs from total 535 collections
At (Expected distribution under H0:no difference in genome distribution between At and Dt)	3(9.04)**	104(94.02)	46(50.02))	135
Dt	12(5.96)	52(61.98)	37 (32.98)	89
	15	156	83	224
X2 test*	<0.01	<0.01	<0.01	
Shared AtDt	29	111	88	157
DD	1	15	5	16
Unallocated	10	0	92	102
Total	55	182	268	499

*: X^2^/df 5 is 60.92;

**: values in the parentheses are the expected values.

### Subgenomic distribution of fiber transcription factors and fiber development genes

In order to dissect the distribution pattern of different groups of fiber genes, the collected 535 fiber development-related genes were divided into three sub-groups: a fiber development group of 259 fiber genes that were highly expressed during fiber development; a regulatory factor group of 103 transcription factors that regulated the expression of the genes during the fiber development; and a marker group of 173 fiber EST-derived markers that are genetically mapped and used to anchor contigs to a virtual integrated genetic map.

**Figure 2:**
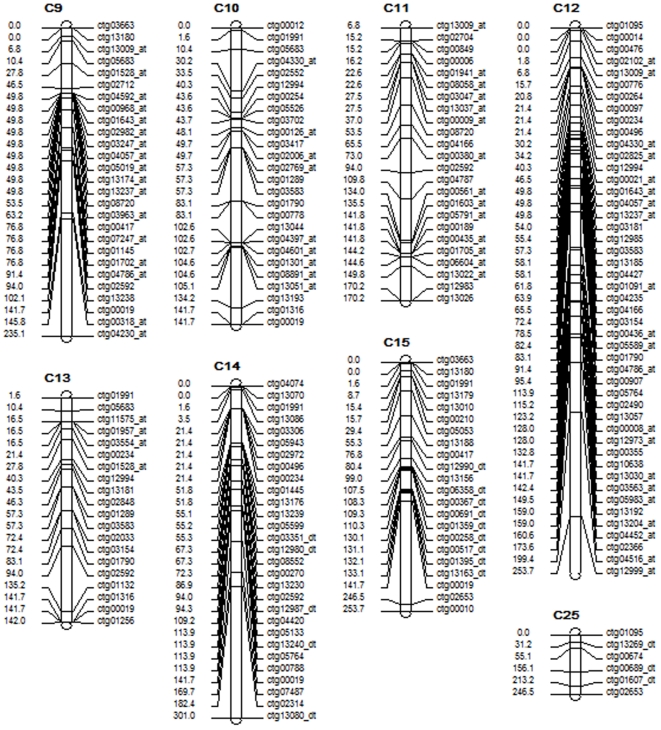
Part of the contig map of the gene distribution patterns between subgenomes At and Dt. Numbers on the left are the genetic distances. BAC-contigs are listed on the right. Ctg stands for contig; the following 5 digits are the contig numbers. If a contig is labeled as “at” after the contig name, it means that this contig is anchored to subgenome At only, same as “dt”. The contig that is not labeled either “at” or “dt” is shared between subgenomes At and Dt. The detailed mapping information is available in the supplemental Figure S1.

A total of 55, 182, and 268 BAC contigs were obtained from 103 transcription factors, 173 SSR-containing fiber ESTs, and 259 fiber development genes, respectively (Table 3, Figure 2.). Among the 268 BAC contigs identified from 259 fiber development genes, 46 BAC contigs were anchored to At subgenome, 37 contigs to Dt subgenome, 88 contigs were shared between At and Dt subgenomes and 92 contigs were not integrated with genetic maps. Similarly, among the 182 BAC contigs, 104 contigs were anchored to At subgenome, 52 to Dt subgenome, and 111 were shared between At and Dt subgenomes. Both results showed that more BAC contigs were anchored to subgenome At than Dt. These results are consistent with the larger genome size of the A genome (1860 Mb), which is twice the size of the D genome (980 Mb) [53]. In contrast, among the 55 BAC contigs identified from 103 transcription factors, only 3 contigs (20%) were anchored to At subgenome, while 12 contigs (80%) were anchored to Dt. Clearly, more transcription factors were anchored in Dt subgenome than those in At subgenome.

## Discussion

### Dt subgenome regulates the expression of fiber genes in At subgenome

Cultivated tetraploid cottons (*G. hirsutum* and *G. barbadense*), possessing both an At and a Dt subgenome, are thought to have formed about 1–2 *MYA*, in the New World, by hybridization between a maternal Old World “A” genome taxon resembling *G. arboreum* and a paternal New World “D” genome taxon resembling *G. raimondii* [10], [12]. Domesticated A diploids were intensively bred and cultivated up until the mid twentieth century. In contrast, none of the D genome diploids, including the presumed Dt subgenome donor, are cultivated because they do not produce spinnable fibers, even though their seeds are pubescent [54]. Although both A genome diploid and AD tetraploid *Gossypium* taxa produce spinnable fibers, and both of them are still planted for fibers by farmers, the yield and quality from domesticated A genome diploids (*G. arboreum* and *G. herbaceum*) are lower than that from AD tetraploid cottons (*G. hirsutum* and *G. barbadense*). A question that arises and needs an answer is why tetraploid cottons consistently have higher yield and quality than the modern descendants of their diploid progenitors. Both human and natural selection pressures do not readily explain why polyploidized tetraploid cottons produce unique modern fiber after the merging of the two diploid genomes A and D [6]. By genetic mapping of 14 RFLP-based fiber QTLs, one conclusion was drawn from an observation that most of QTLs (10 of the 14 QTLs) influencing fiber quality and yield are located on the Dt subgenome [6]. This conclusion was further confirmed by a meta-mapping of 432 QTLs, of which 221 and 184 QTLs were mapped to subgenomes Dt and At, respectively [9]. By dissecting the QTLs based on their function for fiber development, the Dt subgenome contained 112 lint fiber-related QTL *vs.* 84 in the At. When the QTLs were further classified into elongation (EL), fiber color (FC), fiber fineness (FF), fiber length (FL), fiber strength (FS), fiber uniformity (FU), Micronaire (MIC), and short fiber content (SF), all traits except FC and FL have more QTLs on the Dt subgenome than on the At. Comparing this report with previous mapping studies, the difference is that not all kinds of QTLs were located preferably in Dt subgenome than At. Both QTLs of fiber length and fiber color were anchored more in At subgenome than Dt. In our results, when all the BAC contigs from 535 fiber genes/candidates were assigned to their subgenomes, more contigs were anchored to the At subgenome than to the Dt (135 vs. 89) (Table 3). However, when contigs were divided into sub-groups based on their function as transcription factor contigs, fiber gene contigs, and fiber EST contigs, more transcription factor contigs were anchored to the Dt subgenome than the At (12 vs. 3). In contrast, more fiber gene contigs were anchored to subgenome At than Dt (104 vs. 52). Based on our data and previous reports that more fiber QTLs map to subgenome Dt than At, we suggest a functional hypothesis for tetraploid cottons in which the At subgenome in the merged tetraploid genome (*G. hirsutum* or *G. barbadense*) functions similarly to its probable diploid ancestor (*G. arboreum* or *G. herbaceum)* in providing most of the genes for fiber development. On the other hand, the Dt subgenome, with its probable ancestor D genome (*G. raimondii* or *G. gossypioides*) provides more transcription factors that regulate the expression of the fiber genes in the At subgenome. Together with domestication and natural and human biased selection, the regulation of expression of fiber genes in the At subgenome by factors in Dt subgenome has enhanced the expression of the fiber genes, resulting in both the fiber yield and quality improvement.

### Re-analysis of previous results with the hypothesis

The above stated hypothesis would explain previously published results. By aligning all the QTL mapping data from 11 mapping populations, including one diploid and 10 tetraploid interspecific cotton populations, 432 QTLs were anchored to a virtual consensus map by web-based comparison tool cMAP and meta analysis of the QTLs [9]. Distribution of the QTLs between the At and Dt subgenomes was biased, with the Dt subgenome containing more QTLs (211) than the At (184). Considering the fiber-related QTLs, the Dt subgenome contained more lint fiber-related QTLs (112) than the At (84). For comparison, markers from the most saturated genetic map [44] were re-clustered according to the origin of the markers used in the map and only those that were developed from fiber cells were statistically analyzed. Among the 1,749 cDNA markers, 853 markers are from a 7–10 day (fiber elongation period) fiber cell cDNA library of *G. arboreum*. Among 853 fiber ESTs, 419 EST markers were mapped to At subgenome, and 360 EST markers to Dt subgenome, whereas 74 were mapped to the diploid D genome. Apparently, there were more fiber-related ESTs mapped to At subgenome (419) than Dt (360), even though functions of these fiber-related ESTs are unconfirmed as to whether they are specific to fiber development other than they were generated from a fiber cell cDNA library, they are randomly selected from a large number of 46,603 ESTs in a cDNA library and they may represent the fiber elongation genes.

A similar fiber gene/QTL distribution pattern of At and Dt subgenomes was obtained from reanalyzing the fiber EST-SSR and QTL mapping. Of 211 EST-SSR markers, 132 fiber EST-SSR markers were mapped in At subgenome and 79 in Dt subgenome [42]. This result was further confirmed when more fiber EST-SSR markers were added, of which more fiber EST-SSR markers were mapped in At subgenome (207) than in Dt (175) [55]. In contrast with fiber EST distribution, a different QTL distribution pattern was obtained from the same group. Of the 25 major QTLs (LOD >3.0) and 28 putative QTLs (2.0 < LOD < 3.0) for fiber quality and yield components that were identified, more QTLs mapped to the Dt subgenome (43) than At (10) [56]. Comparing the results from fiber EST mapping with that from QTL mapping, more fiber ESTs were anchored in At subgenome than Dt, whereas more QTLs were mapped in Dt subgenome than At subgenome. Comparing both the QTL and fiber-EST mapping data reported from the same group, the results were consistent with our hypothesis that At subgenome contains more fiber development genes than Dt, whereas the Dt subgenome contributes more QTL loci/regulation factors than At if one QTL represents one locus of a regulation factor that controls the quantitative traits of cotton fiber.

As to genetic mapping of transcription factors, only one report was found [57]. Four of the six MYB transcription factors were anchored in five chromosomes via deletion analysis and linkage mapping, in which three were mapped in Dt subgenome and two were mapped in At subgenome [57]. In addition, nucleotide diversity analysis indicated that the six MYB loci evolved more quickly in the Dt than At subgenome of tetraploid cotton. Even though a very limited number of transcription factors were mapped, they were randomly chosen as a sample and may represent the characteristics of the genome. Mapping results on both fiber ESTs and fiber transcription factors suggested that At subgenome contributes more fiber development genes and Dt subgenome contributes more transcription factors.

### Evolution of fiber genes in tetraploid cottons

The AtDt polyploidization (1–2 MYA) of two differentiated genomes (AA and DD), which diverged from a common ancestor between 6 and 11 MYA, in a common nucleus, has been accompanied by myriad genomic alteration and gene expression changes [58], [59]. Expression changes certainly happened after the merger of the two genomes. Less is known of how the homoeologous genes from subgenomes At and Dt have changed. Study on function diversification of duplicated copies of genes revealed that gene copies from genome duplications (polyploidizations) experience different fates during their evolution including gene loss, subfunctionalization [60], and neofunctionalization [47], [61]. This raises the possibility that differential evolution of homoeologous fiber-related genes duplicated by polyploid formation (Cronn *et al.* 1999) is partly responsible for modern cotton fiber quality. Data from a SNP-specific microarray investigation showed possible transcription-level evidence recruiting D-genome homoeolog followed by polyploidy formation and suggested two possibilities for superior cultivated tetraploid cotton: one is through generation of novel functional genes by polyploidization of the two genomes; the other one is the enhancement of expression levels of the genes, especially D-genome expression was preferentially enhanced under human selection pressure [59]. Our data is closer to the second option than the first one, because there is no current evidence to support that there are novel genes generated from polyploidization. We suggest that both the expression levels of the genes in At and factors in Dt were enhanced after polyploidization, based upon the observed phenomenon of more regulation factors in Dt subgenome and more fiber genes in At, and previous results from SNP-specific microarray investigations [25], [35]. However, their expression was enhanced at different times. First, transcription factors enhance their expression level in Dt subgenome, and then as regulation factors, they regulate the expression level of fiber genes in At, and as the final step the fiber genes in At were enhanced. As to the shared transcription factors between At and Dt subgenomes, these exhibit subfunctionalization, in that two copies partition the ancestral function [47]. The few transcription factors that were mapped only in At subgenome are evidence of neofunctionalization, in that genes mutated into a function that was not present pre-polyploidization [47], but currently play regulatory roles in the corresponding homoeologous transcription factors in Dt, and regulate the expression of the fiber genes in At. These speculations await more functional investigation, but the distribution data from our report and expression data from SNP-specific microarray may provide useful clues in this regard.

The integrated map of fiber development genes would provide a framework to clone individual full-length fiber genes, to elucidate the physiological mechanisms of the fiber differentiation, elongation, and maturation, and to systematically study the functional network of interacting genes during the process of fiber development in tetraploid cotton.

### Reliability of the methodology vs. materials used in the study

This study began with collecting sequences of cotton fiber development genes and transcription factors that were previously published. Most of these studies focused on fiber development genes [18], [25], [28], [34], [37], [41], [42], [48], [62], [63], [64], [65], [66], [67], [68], [69]. There is more fiber development gene information accumulated from the four fiber development stages, fiber initiation, elongation, second wall deposition, and maturation than from the study of transcription factors. Compared with large-scale EST sequencing, individual studies have concentrated more on transcription factors, especially using comparative genomics with Arabidopsis [29], [35], [57], [70], [71], [72], [73]. The sequence collection reflected the research focuses in that 432 of the 535(80.7%) collected sequences are fiber development genes. In contrast, only 103(19.7%) collected sequences are transcription factors. This biased collection could affect our results and mislead our conclusion. As to the origin/source of the fiber development genes and transcription factors between the two subgenomes At and Dt, 28 of the 31 cDNA libraries were constructed from tetraploid cotton which indicated that genes and transcription factors in At and Dt have same chances from these libraries which comprised of 38% of the total ESTs[74]. The remaining three EST libraries were derived from two diploids (one library from 7–10 dpa of A-genome *G. arboreum* and two libraries of D-genome *G. raimondii*), which comprised 24 and 38% of the total number of ESTs, respectively [75]. Thus, more genes and factors were collected from two diploid genomes, A and D (62%) than from the At and Dt subgenomes (38%), even though genomes A and D are the ancestor donors of subgenomes At and Dt. This fact has biased EST development in the cotton research community and may have affected this study, too. As a correction to above biased EST development and biased sequence collection, all the three BAC/BIBAC libraries used in this study were constructed from tetraploid cottons that have an equal chance to identify gene distributions between subgenomes At and Dt [76], [77], [78]. The Overgo approach to anchor genes and genetic markers to BACs was applied in other organisms, and it has been proven that it is a reliable method even though the size of Overgo probes is only 44 base pairs [79], [80]. The reason that Overgo hybridization strategy was successfully used in the construction of integrated genetic and physical maps was that Overgo probes were designed based on unique sequences from the genome sequences, not from repetitive sequences. This fact minimized the number of positive clones dramatically. Secondly, hybridization temperature was strictly set at 65°C which makes sure that only > 99% matching probes could anneal with the clones. As to more positive BAC clones were obtained when the two-round hybridization method was used, more stringent cutoff (10e−20) was used to assemble the BAC contigs to minimize the false positive BACs in the contig. In general, the methods used in this study, including, sequence collection, BAC high-density filter preparation, Overgo hybridization, and anchoring the BAC contigs to individual chromosomes, are reliable methods. With the new technology of genome sequencing, this method will be further validated and confirmed in the near future. The strategy used in this study and results derived from this research, from collecting previous published sequences, summarizing and clustering them, anchoring them to chromosomes, provide a platform for structure and function genomics to study these genes systematically on large scales.

## Materials and Methods

### Collection of fiber development genes/transcription factors and assembly of sequence contigs

In this study, four groups of fiber development genes were identified: first group, ESTs that were generated from fiber cell, investigated by microarray or RT-PCR function profiling, and confirmed enrichment expression during fiber development, including up-regulation and down-regulation genes [18], [48]; second group, individual genes that were not only confirmed by microarray expression, but also were transformed to Arabidopsis or tobacco to verify their function by complementation test [35], [67], [81], [82]; third group, transcription factors, including MYB family, AP2/EREBP family, and GARP-G2-like transcription factors [36], [38]; and last group, genetically mapped fiber-derived EST gene candidates [40], [41], [44]. All four groups of genes were collected from previous publications and their sequences were downloaded based on the accession numbers from NCBI http://www.ncbi.nlm.nih.gov/or collected from dissertation text (Dr. Hassan's dissertation, Texas A&M University library). The detailed list of all the genes/gene candidates was summarized in supplemental Table S1.

Sequenced contigs were assembled using “Sequencher” version 4.2 (Gene Codes Corporation, Ann Arbor USA) in order to minimize the redundancy of the sequences or to get longer continuous partial overlapped sequences with the parameters set at minimum match 90%, overlap 30 base pairs, and default data algorithm.

### BAC and BIBAC libraries

Three bacterial artificial chromosome (BAC) and binary bacterial artificial chromosome (BIBAC) libraries from tetraploid cottons were used in this study. Of them, two TM-1 BAC/BIBAC libraries were constructed by USDA-ARS Crop Germplasm Research Unit in collaboration with Texas A&M University [76], [77]. The *Bam*HI library was cloned into a BAC-based binary plant transformation vector (BIBAC vector; pCLD04541) while the *Hin*dIII library was cloned using a standard BAC vector (pBeloBAC11). The *Bam*HI library contains 76,800 clones with an average insert size of 130 kb, and covering 4.4 haploid genome equivalents. The *Hin*dIII BAC library contains 76,800 clones with an average insert size of 152 kb. The third BAC library used in this study was constructed from the cotton cultivar Maxxa using *Hin*dIII, at the Clemson University Genomics Institute [83], and it contains 129,024 clones with an average insert size of 137 kb. The libraries provided ∼8X genome coverage. The Maxxa BAC library was partially end-sequenced (∼50,000 reads) and mined for putative SSRs[84]. About 2,600 BAC clones associated with SSR markers were obtained from the library and included in this study. In total, 6.7 x genome equivalent BAC clones were screened for this study (Table 4). High-density colony filter arrays were prepared using a Biomek 2000 robotic workstation equipped with a high-density replicating system (HDR) (Beckman Coulter Inc., Fullerton, California). Each filter was gridded with 1,536 BAC clones using a 4×4 matrix pattern with a 384-pin HDR tool. Filters were incubated and processed as described by[50].

**Table 4:** BAC/BIBAC libraries used in the study and number of fingerprinted clones (Genome size of 2118 Mb was used per [78]).

Genotype	Mean insert size	No. of clones	Genome coverage	Vector type	Cloning site	No. of fingerprinted clones and genome coverage
TM-1 libraries						
TM-1	152 kb	76,800	5.5 x	pECBAC1	*Hind*III	24,576 (1.8 x)
TM-1	132 kb	76,800	4.7 x	pCLD04541	*Bam*HI	76,800 (4.7 x)
Maxxa library						
Maxxa	137 kb	2,603	0.2 x	pCUGI-1	*Hind*III	2,603 (0.2 x)
Total	141 kb	156,203	10.4 x			103,979 (6.7 x)

### Two-round hybridization to screen BAC libraries

Two-round hybridization method was used to screen BAC libraries. The first round was to screen BACs libraries using Overgo probes (44 base pair long) and protocol for BAC-filters preparation, Overgo probe design, pre-hybridization, and hybridization were same as [50]. The second-round was to re-screen the three BAC libraries with representative positive BACs selected from individual contigs and all singleton BACs after automatic identification of contigs from the first-round hybridization. In detail, positive BACs from the first-round hybridization were automatically assembled into contigs by using a cutoff parameter of 10e−12 and band tolerance of 0.2 bp, which is the resolution of the 36 cm capillary of the Sequencer ABI3100. One representative BAC from each small contig, two BACs from each large contig, together with all the singletons, were used as BAC pool DNA to screen the BAC libraries again for better coverage. As expected, more BACs will be identified because of the homologs between At and Dt subgenomes. Contigs obtained by hybridization in this report were compared and verified with those from the genome-wide physical contig map (Xu et al., in preparation). All the positive BACs identified by the two-round hybridization were compared and verified with the contigs that contained the BACs indentified by the first-round hybridization and were assembled from the whole genome physical map of the tetraploid cotton. Only the shared BACs from above comparisons were put together as an FPC project and were assembled into contigs.

### BAC fingerprinting, contig assembly, and contig sorting to cotton chromosomes

BAC DNA isolation, fingerprinting, contig assembly were same as [50] except that DNA fingerprinting raw data were edited using “GenoProfiler” [51] which is different from the one (ABI-to-FPC, unpublished) used for whole genome physical map of tetraploid cotton. The reason that “GenoProfiler” software package was used to edit the fingerprinting raw data is to compare the contig assembly results obtained from different editing methods. Band size text file generated by “GenoProfiler” was copied to the folder “Size” under the FPC folder for contig assembly [52].

Contigs were anchored to chromosomes by comparing the genetic markers and BAC-end sequences with STS at expected values (<e−30). Genetic markers were anchored into contig map by Overgo hybridization. For verification and development of new markers, BAC-ends were sequenced from the representative BACs selected from each contigs. BAC-end sequences from these contigs also were used to blast against all the STSs mapped in the integrated genetic and physical map of tetraploid cotton at an expected value of 1e−30.

### STS database setup and Blast analysis

**Figure 3:**
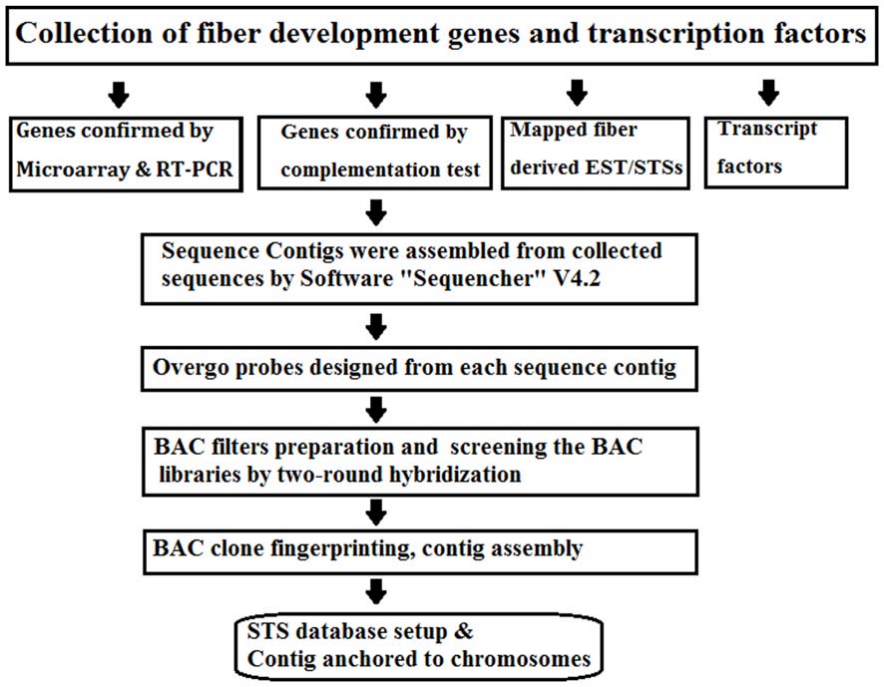
Workflow of this research. Schematic flowchart of anchoring the fiber development genes and transcript factors into subgenomes At and Dt.

Non-redundant STSs were collected from 27 genetic and physical map related publications and their sequences were downloaded from NCBI. BAC-end and BAC-sub clone sequences were generated from BAC-based physical contigs, together with public STSs, were used to set up an STS database for blast analysis. The Blast program “blastall” was downloaded from NCBI and used to annotate the sequences. The criterion for sequence match, expected value E = 1e−30, was used to perform the blast analysis. Assembled BAC contigs with either fiber development genes or transcription factors, or both were anchored on subgenomes At or Dt by identifying 1) genetic markers in the contig; 2) BAC-end sequences with STS sequences. The entire workflow was summarized in figure 3.

## Supporting Information

Figure S1 BAC Contig map of fiber development genes and transcription factors
(0.14 MB PDF)

Supplemental Table S1 (0.55 MB XLS)

Supplemental Table S2 (0.13 MB XLS)
