# Supplementary material for: Polyploidization Altered Gene Functions in Cotton (Gossypium spp.)
Source: PLoS One. 2010 Dec 16;5(12):e14351. doi: 10.1371/journal.pone.0014351 (PMC3002935; doi:10.1371/journal.pone.0014351)
Supplement: Figure S1 — BAC Contig map of fiber development genes and transcription factors (0.14 MB PDF) [file pone.0014351.s001.pdf]

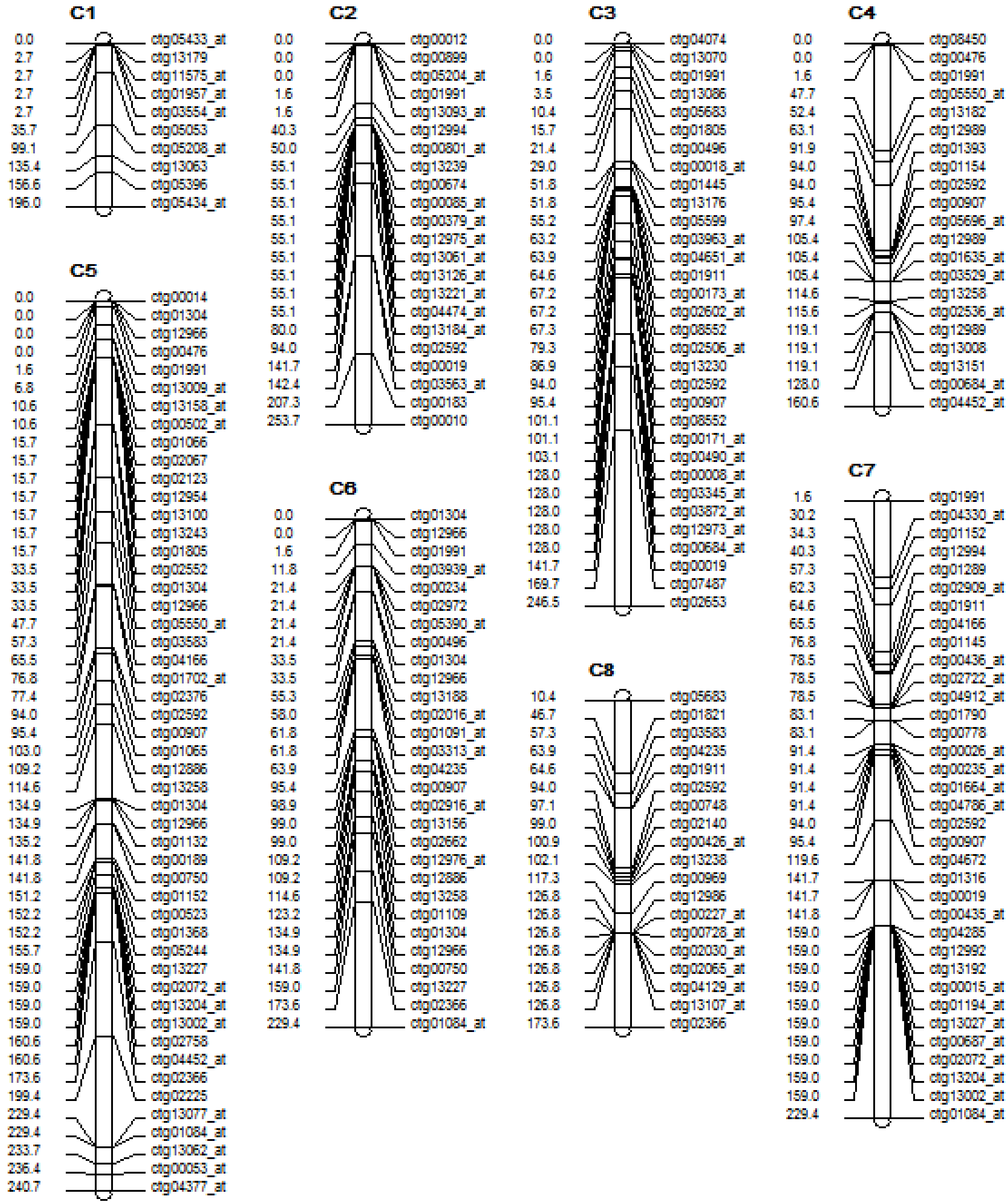

C1
0.0 ctg05433_at
2.7 ctg13179
2.7 ctg11575_at
2.7 ctg01957_at
2.7 ctg03554_at
35.7 ctg05053
99.1 ctg05208_at
135.4 ctg13063
156.6 ctg05396
196.0 ctg05434_at
C2
0.0 ctg00012
0.0 ctg00899
0.0 ctg05204_at
1.6 ctg01991
1.6 ctg13093_at
40.3 ctg12994
50.0 ctg00801_at
55.1 ctg13239
55.1 ctg00674
55.1 ctg00085_at
55.1 ctg00379_at
55.1 ctg12975_at
55.1 ctg13061_at
55.1 ctg13126_at
55.1 ctg13221_at
55.1 ctg04474_at
80.0 ctg13184_at
94.0 ctg02592
141.7 ctg00019
142.4 ctg03563_at
207.3 ctg00183
253.7 ctg00010
C3
0.0 ctg04074
0.0 ctg13070
1.6 ctg01991
3.5 ctg13086
10.4 ctg05683
15.7 ctg01805
21.4 ctg00496
29.0 ctg00018_at
51.8 ctg01445
51.8 ctg13176
55.2 ctg05599
63.2 ctg03963_at
63.9 ctg04651_at
64.6 ctg01911
67.2 ctg00173_at
67.2 ctg02602_at
67.3 ctg08552
79.3 ctg02506_at
86.9 ctg13230
94.0 ctg02592
95.4 ctg00907
101.1 ctg08552
101.1 ctg00171_at
103.1 ctg00490_at
128.0 ctg00008_at
128.0 ctg03345_at
128.0 ctg03872_at
128.0 ctg12973_at
128.0 ctg00684_at
141.7 ctg00019
169.7 ctg07467
246.5 ctg02653
C4
0.0 ctg08450
0.0 ctg00476
1.6 ctg01991
47.7 ctg05550_at
52.4 ctg13182
63.1 ctg12989
91.9 ctg01393
94.0 ctg01154
94.0 ctg02592
95.4 ctg00907
97.4 ctg05696_at
105.4 ctg12989
105.4 ctg01635_at
105.4 ctg03529_at
114.6 ctg13258
115.6 ctg02536_at
119.1 ctg12989
119.1 ctg13008
119.1 ctg13151
128.0 ctg00684_at
160.6 ctg04452_at
C5
0.0 ctg00014
0.0 ctg01304
0.0 ctg12966
0.0 ctg00476
1.6 ctg01991
6.8 ctg13009_at
10.6 ctg13158_at
10.6 ctg00502_at
15.7 ctg01066
15.7 ctg02067
15.7 ctg02123
15.7 ctg12954
15.7 ctg13100
15.7 ctg13243
15.7 ctg01805
33.5 ctg02552
33.5 ctg01304
33.5 ctg12966
47.7 ctg05550_at
57.3 ctg03583
65.5 ctg04166
76.8 ctg01702_at
77.4 ctg02376
94.0 ctg02592
95.4 ctg00907
103.0 ctg01065
109.2 ctg12886
114.6 ctg13258
134.9 ctg01304
134.9 ctg12966
135.2 ctg01132
141.8 ctg00189
141.8 ctg00750
151.2 ctg01152
152.2 ctg00523
152.2 ctg01368
155.7 ctg05244
159.0 ctg13227
159.0 ctg02072_at
159.0 ctg13204_at
159.0 ctg13002_at
160.6 ctg02758
160.6 ctg04452_at
173.6 ctg02366
199.4 ctg02225
229.4 ctg13077_at
229.4 ctg01084_at
233.7 ctg13062_at
236.4 ctg00053_at
240.7 ctg04377_at
C6
0.0 ctg01304
0.0 ctg12966
1.6 ctg01991
11.8 ctg03939_at
21.4 ctg00234
21.4 ctg02972
21.4 ctg05390_at
21.4 ctg00496
33.5 ctg01304
33.5 ctg12966
55.3 ctg13188
58.0 ctg02016_at
61.8 ctg01091_at
61.8 ctg03313_at
63.9 ctg04235
95.4 ctg00907
98.9 ctg02916_at
99.0 ctg13156
99.0 ctg02662
109.2 ctg12976_at
109.2 ctg12886
114.6 ctg13258
123.2 ctg01109
134.9 ctg01304
134.9 ctg12966
141.8 ctg00750
159.0 ctg13227
173.6 ctg02366
229.4 ctg01084_at
C7
1.6 ctg01991
30.2 ctg04330_at
34.3 ctg01152
40.3 ctg12994
57.3 ctg01289
62.3 ctg02909_at
64.6 ctg01911
65.5 ctg04166
76.8 ctg01145
78.5 ctg00436_at
78.5 ctg02722_at
78.5 ctg04912_at
83.1 ctg01790
83.1 ctg00778
91.4 ctg00026_at
91.4 ctg00235_at
91.4 ctg01664_at
91.4 ctg04786_at
94.0 ctg02592
95.4 ctg00907
119.6 ctg04672
141.7 ctg01316
141.7 ctg00019
141.8 ctg00435_at
159.0 ctg04285
159.0 ctg12992
159.0 ctg13192
159.0 ctg00015_at
159.0 ctg01194_at
159.0 ctg13027_at
159.0 ctg00687_at
159.0 ctg02072_at
159.0 ctg13204_at
159.0 ctg13002_at
229.4 ctg01084_at
C8
10.4 ctg05683
46.7 ctg01821
57.3 ctg03583
63.9 ctg04235
64.6 ctg01911
94.0 ctg02592
97.1 ctg00748
99.0 ctg02140
100.9 ctg00426_at
102.1 ctg13238
117.3 ctg00969
126.8 ctg12986
126.8 ctg00227_at
126.8 ctg00728_at
126.8 ctg02030_at
126.8 ctg02065_at
126.8 ctg04129_at
126.8 ctg13107_at
173.6 ctg02366

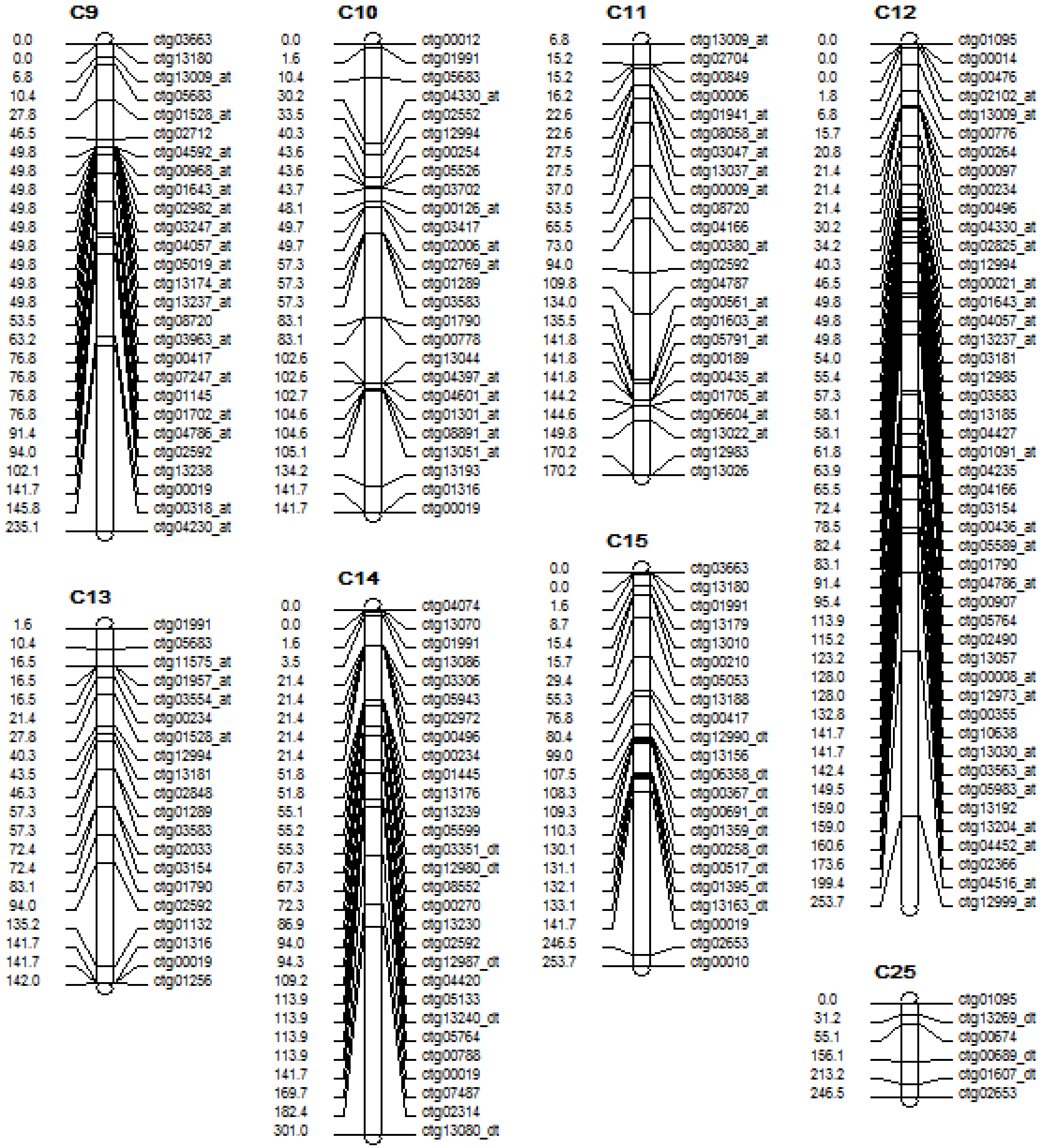
C9
C10
C11
C12
C13
C14
C15
C25

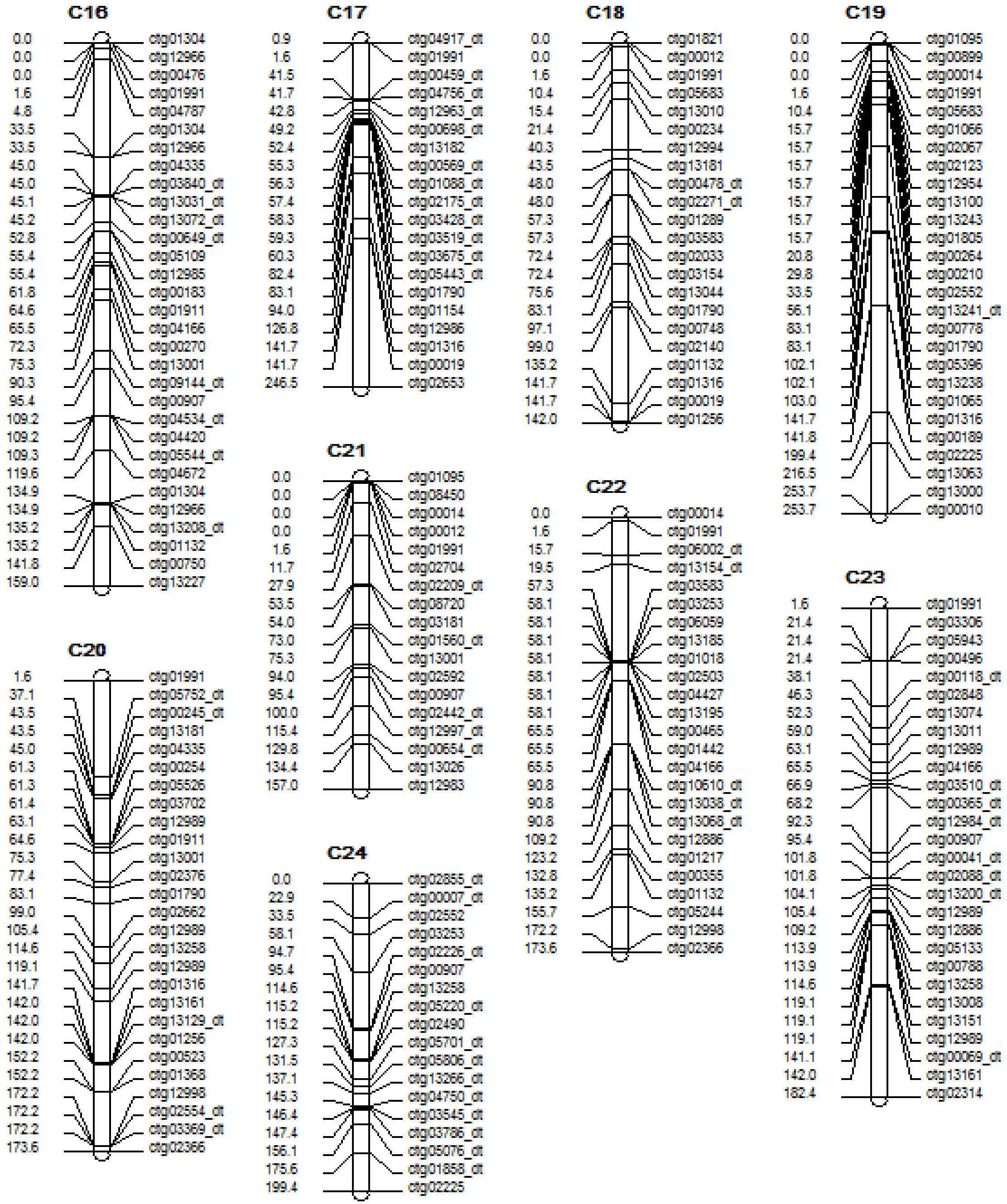
C16
0.0 ctg01304
0.0 ctg12966
0.0 ctg00476
1.6 ctg01991
4.8 ctg04787
33.5 ctg01304
33.5 ctg12966
45.0 ctg04335
45.0 ctg03840_dt
45.1 ctg13031_dt
45.2 ctg13072_dt
52.8 ctg00649_dt
55.4 ctg05109
55.4 ctg12985
61.8 ctg00183
64.6 ctg01911
65.5 ctg04166
72.3 ctg00270
75.3 ctg13001
90.3 ctg09144_dt
95.4 ctg00907
109.2 ctg04534_dt
109.2 ctg04420
109.3 ctg05544_dt
119.6 ctg04672
134.9 ctg01304
134.9 ctg12966
135.2 ctg13208_dt
135.2 ctg01132
141.8 ctg00750
159.0 ctg13227
C17
0.9 ctg04917_dt
1.6 ctg01991
41.5 ctg00459_dt
41.7 ctg04756_dt
42.8 ctg12963_dt
49.2 ctg00698_dt
52.4 ctg13182
55.3 ctg00569_dt
56.3 ctg01088_dt
57.4 ctg02175_dt
58.3 ctg03428_dt
59.3 ctg03519_dt
60.3 ctg03675_dt
82.4 ctg05443_dt
83.1 ctg01790
94.0 ctg01154
126.8 ctg12986
141.7 ctg01316
141.7 ctg00019
246.5 ctg02653
C18
0.0 ctg01821
0.0 ctg00012
1.6 ctg01991
10.4 ctg05683
15.4 ctg13010
21.4 ctg00234
40.3 ctg12994
43.5 ctg13181
48.0 ctg00478_dt
48.0 ctg02271_dt
57.3 ctg01289
57.3 ctg03583
72.4 ctg02033
72.4 ctg03154
75.6 ctg13044
83.1 ctg01790
97.1 ctg00748
99.0 ctg02140
135.2 ctg01132
141.7 ctg01316
141.7 ctg00019
142.0 ctg01256
C19
0.0 ctg01095
0.0 ctg00899
0.0 ctg00014
1.6 ctg01991
10.4 ctg05683
15.7 ctg01066
15.7 ctg02067
15.7 ctg02123
15.7 ctg12954
15.7 ctg13100
15.7 ctg13243
15.7 ctg01805
20.8 ctg00264
29.8 ctg00210
33.5 ctg02552
56.1 ctg13241_dt
83.1 ctg00778
83.1 ctg01790
102.1 ctg05396
102.1 ctg13238
103.0 ctg01065
141.7 ctg01316
141.8 ctg00189
199.4 ctg02225
216.5 ctg13063
253.7 ctg13000
253.7 ctg00010
C20
1.6 ctg01991
37.1 ctg05752_dt
43.5 ctg00245_dt
43.5 ctg13181
45.0 ctg04335
61.3 ctg00254
61.3 ctg05526
61.4 ctg03702
63.1 ctg12989
64.6 ctg01911
75.3 ctg13001
77.4 ctg02376
83.1 ctg01790
99.0 ctg02662
105.4 ctg12989
114.6 ctg13258
119.1 ctg12989
141.7 ctg01316
142.0 ctg13161
142.0 ctg13129_dt
142.0 ctg01256
152.2 ctg00523
152.2 ctg01368
172.2 ctg12998
172.2 ctg02554_dt
172.2 ctg03369_dt
173.6 ctg02366
C21
0.0 ctg01095
0.0 ctg08450
0.0 ctg00014
0.0 ctg00012
1.6 ctg01991
11.7 ctg02704
27.9 ctg02209_dt
53.5 ctg08720
54.0 ctg03181
73.0 ctg01560_dt
75.3 ctg13001
94.0 ctg02592
95.4 ctg00907
100.0 ctg02442_dt
115.4 ctg12997_dt
129.8 ctg00654_dt
134.4 ctg13026
157.0 ctg12983
C22
0.0 ctg00014
1.6 ctg01991
15.7 ctg06002_dt
19.5 ctg13154_dt
57.3 ctg03583
58.1 ctg03253
58.1 ctg06059
58.1 ctg13185
58.1 ctg01018
58.1 ctg02503
58.1 ctg04427
58.1 ctg13195
65.5 ctg00465
65.5 ctg01442
65.5 ctg04166
90.8 ctg10610_dt
90.8 ctg13038_dt
90.8 ctg13068_dt
109.2 ctg12886
123.2 ctg01217
132.8 ctg00355
135.2 ctg01132
155.7 ctg05244
172.2 ctg12998
173.6 ctg02366
C23
1.6 ctg01991
21.4 ctg03306
21.4 ctg05943
21.4 ctg00496
38.1 ctg00118_dt
46.3 ctg02848
52.3 ctg13074
59.0 ctg13011
63.1 ctg12989
65.5 ctg04166
66.9 ctg03510_dt
68.2 ctg00365_dt
92.3 ctg12984_dt
95.4 ctg00907
101.8 ctg00041_dt
101.8 ctg02088_dt
104.1 ctg13200_dt
105.4 ctg12989
109.2 ctg12886
113.9 ctg05133
113.9 ctg00788
114.6 ctg13258
119.1 ctg13008
119.1 ctg13151
119.1 ctg12989
141.1 ctg00069_dt
142.0 ctg13161
182.4 ctg02314
C24
0.0 ctg02855_dt
22.9 ctg00007_dt
33.5 ctg02552
58.1 ctg03253
94.7 ctg02226_dt
95.4 ctg00907
114.6 ctg13258
115.2 ctg05220_dt
115.2 ctg02490
127.3 ctg05701_dt
131.5 ctg05806_dt
137.1 ctg13266_dt
145.3 ctg04750_dt
146.4 ctg03545_dt
147.4 ctg03786_dt
156.1 ctg05076_dt
175.6 ctg01858_dt
199.4 ctg02225

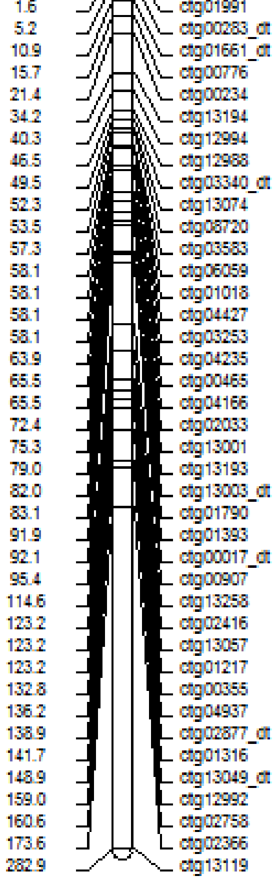
1.6 ctg01991
5.2 ctg00283_dt
10.9 ctg01661_dt
15.7 ctg00776
21.4 ctg00234
34.2 ctg13194
40.3 ctg12994
46.5 ctg12968
49.5 ctg03340_dt
52.3 ctg13074
53.5 ctg08720
57.3 ctg03583
58.1 ctg06059
58.1 ctg01018
58.1 ctg04427
58.1 ctg03253
63.9 ctg04235
65.5 ctg00465
65.5 ctg04166
72.4 ctg02033
75.3 ctg13001
79.0 ctg13193
82.0 ctg13003_dt
83.1 ctg01790
91.9 ctg01393
92.1 ctg00017_dt
95.4 ctg00907
114.6 ctg13258
123.2 ctg02416
123.2 ctg13057
123.2 ctg01217
132.8 ctg00355
136.2 ctg04937
138.9 ctg02877_dt
141.7 ctg01316
148.9 ctg13049_dt
159.0 ctg12992
160.6 ctg02758
173.6 ctg02366
282.9 ctg13119
